# Supplementary material for: Volatile Chemical Variation of Essential Oils and Their Correlation with Insects, Phenology, Ontogeny and Microclimate: Piper mollicomum Kunth, a Case of Study
Source: Plants (Basel). 2022 Dec 15;11(24):3535. doi: 10.3390/plants11243535 (PMC9785739; doi:10.3390/plants11243535)
Supplement: Supplementary file 1 [file plants-11-03535-s001.zip › plants-2000905-supplementary.pdf]

Table S1. Main chemical constituents of the essential oils from stages 1, 2 and 3 of the reproductive organ of *Piper mollicomum* Kunth from September 2020 to January 2021.

Compounds[a]	N_o. CAS	RIC alc	RI_{Lit}	Relative Percentage %														
				Stage 1					Stage 2					Stage 3				
				Sep	Oct	Nov	Dec	Jan	Sep	Oct	Nov	Dec	Jan	Sep	Oct	Nov	Dec	Jan
α-pinene*	7785-26-4	938	939	18.22	-	10.55	8.58	12.96	18.22	-	9.35	7.04	13.59	-	4.29	2.77	3.50	2.25
camphene*	79-92-5	947	954	25.41	-	-	-	-	-	-	-	-	-	-	7.17	-	-	-
myrcene*	123-35-3	963	974	-	-	1.67	1.15	3.27	-	-	1.25	1.65	2.66	-	1.13	1.27	0.92	1.55
β-pinene*	127-91-3	981	980	-	-	17.13	6.39	15.84	-	-	8.15	6.18	18.19	-	3.16	6.80	8.13	2.42
limonene*	5989-27-5	1028	1029	8.39	17.85	-	-	-	9.17	20.07	-	-	-	14.58	7.44	-	-	-
1,8-cineole**	470-82-6	1032	1031	8.22	-	26.08	32.62	13.51	-	-	44.92	33.02	16.50	-	0.16	51.28	22.93	33.75
Z-β-ocimene*	3338-55-4	1043	1037	3.29	-	-	-	3.18	-	-	-	1.71	0.08	0.73	0.74	-	4.35	0.10
E-β-ocimene*	3779-61-1	1049	1050	-	-	-	-	-	-	-	-	-	-	2.24	2.10	-	-	-
camphor**	464-49-3	1136	1141	-	2.01	-	-	-	-	-	-	-	-	-	11.45	-	-	-
α-terpineole**	98-55-5	1187	1186	-	1.49	3.46	3.96	1.21	-	-	3.39	4.84	2.24	-	0.86	5.61	1.66	3.32
δ-terpineole**	7299-42-5	1188	1186	-	-	-	1.75	0.17	-	-	-	-	1.92	-	-	-	-	1.53
linalool**	78-70-6	1193	1198	29.24	44.29	-	6.11	2.43	29.24	46.21	4.15	-	10.34	62.02	30.27	2.48	8.46	21.60
β-elemene***	33880-83-0	1377	1389	-	-	-	1.20	0.06	-	-	-	0.35	-	-	-	-	-	0.08
E-caryophyllene***	87-44-5	1421	1419	5.36	2.68	-	1.44	0.19	4.42	2.76	-	2.49	-	2.12	3.47	2.77	0.44	0.13
α-humulene***	6753-98-6	1447	1456	-	-	-	-	0.16	-	2.42	-	0.95	0.03	-	2.49	-	2.50	1.34
β-selinene***	17066-67-0	1491	1490	-	-	-	0.24	-	-	-	-	0.75	-	2.39	0.42	-	-	0.14
E-nerolidol****	40716-66-3	1567	1563	2.28	2.90	0.20	0.77	0.14	2.28	-	-	1.46	0.12	5.91	2.92	-	2.66	0.03
aromadendrene***	66105-35-9	1633	1639	-	-	-	-	-	-	-	-	-	-	-	0.86	-	-	-
caryophyllene acetate****	32214-91-8	1703	1701	-	-	2.07	0.43	-	-	-	-	0.26	0.04	-	-	-	0.37	0.50

eupatoriochromene*****	19013-03-7	1732	1761	8.55	16.13	11.85	15.26	2.98	6.35	14.99	8.41	6.55	0.13	5.91	13.21	9.01	15.65	4.57
benzyl benzoate*****	120-51-4	1778	1762	-	-	-	2.00	-	-	-	-	0.08	0.38	-	-	-	0.21	-
**Yield (%)**				**0.29**	**0.01**	**1.12**	**0.06**	**0.01**	**0.13**	**0.09**	**0.05**	**0.03**	**0.12**	**0.09**	**0.16**	**0.10**	**0.07**	**0.04**
**Total of Identified Compounds (%)**				**99.96**	**85.66**	**73.47**	**86.46**	**79.56**	**99.97**	**84.06**	**85.20**	**86.33**	**66.37**	**84.30**	**99.07**	**88.85**	**83.82**	**87.66**

RI_{calc} = Calculated retention index (column HP-5MS); RI_{lit} = Literature retention index (Adams. 2009); [a]All compounds were identified by GC/MS and GC/FID according to the experimental; Sep-September; Oct–October; Nov-November; Dec-December; Jan-January. Retention times (Rt) were measured in minutes without correction, and the relative percentage of each compound was determined by the signal area (2,3,55,167,168]. The RIs were calculated from the results of the analysis of a homologous series of saturated aliphatic hydrocarbons (C_8-C_{28}, Sigma-Aldrich, Brazil), performed in the same column and conditions used for analysis by GC/FID. *Non-oxygenated monoterpenes; **Oxygenated monoterpenes; ***Non-oxygenated sesquiterpenes; ****Oxygenated sesquiterpenes; *****Other compounds. For this analysis, we highlight all the constituents of the EOs of *P. mollicomum* that were observed at least once in the periods and stages of the analyzed reproductive organs.

Table S2. Main chemical constituents of essential oils from phases 4 and 5 of the development of the reproductive organ of *Piper mollicomum* Kunth from September 2020 to January 2021.

**Compounds**	**No. CAS**	**RI_{Calc}**	**RI_{Lit}**	**Relative Percentage %**							**Spearman Correlation (r^2)**
				**Stage 4**					**Stage 5**		
				**Set**	**Out**	**Nov**	**Dez**	**Jan**	**Dez**	**Jan**	
α-pinene*	7785-26-4	938	939	-	-	6.80	14.70	-	6.95	4.71	**-0.878**
camphene*	79-92-5	947	954	-	6.38	-	-	-	-	-	-0.108
myrcene*	123-35-3	963	974	-	-	-	-	-	2.24	-	**-0.799**
β-pinene*	127-91-3	981	980	-	-	5.40	3.19	2.49	5.52	6.01	-0.320
limonene*	5989-27-5	1028	1029	6.30	3.12	-	-	-	-	-	-0.303
1,8-cineole**	470-82-6	1032	1031	-	-	58.78	23.08	49.32	26.28	47.72	-0.024
Z-β-ocimene*	3338-55-4	1043	1037	0.81	-	-	0.77	1.67	1.28	0.20	-0.031
E-β-ocimene*	3779-61-1	1049	1050	3.21	-	-	-	-	-	0.89	0.221
camphor**	464-49-3	1136	1141	-	11.26	-	-	-	-	0.16	0.068
α-terpineole**	98-55-5	1187	1186	-	4.92	6.06	5.03	3.94	2.12	4.17	0.010
δ-terpineole**	7299-42-5	1188	1186	-	-	-	-	-	-	-	-0.381
linalool**	78-70-6	1193	1198	73.14	51.96	4.07	4.46	7.54	10.47	6.78	-0.132
β-elemene***	33880-83-0	1377	1389	-	-	-	-	-	0.15	-	-0.216
E-caryophyllene***	87-44-5	1421	1419	2.48	3.37	-	-	0.81	2.45	0.70	-0.287

α-humulene***	6753-98-6	1447	1456	-	-	-	0.25	0.33	1.78	1.19	0.097
β-selinene***	17066-67-0	1491	1490	2.01	-	-	-	0.14	0.34	-	0.057
E-nerolidol****	40716-66-3	1567	1563	2.25	-	-	0.13	1.70	2.49	0.28	-0.269
aromadendrene***	66105-35-9	1633	1639	1.06	-	-	-	-	-	-	0.108
caryophyllene acetate****	32214-91-8	1703	1701	-	-	-	1.13	0.20	0.58	0.24	-0.018
eupatoriochromene*****	19013-03-7	1732	1761	4.84	5.63	6.84	32.92	3.71	9.65	2.37	**-0.755**
benzyl benzoate*****	120-51-4	1778	1762	-	-	-	-	-	1.03	0.16	-0.016
**Yield (%)**				**0.08**	**0.11**	**0.11**	**0.01**	**0.57**	**1.47**	**1.20**	
**Total of Identified Compounds (%)**				**96.15**	**98.18**	**92.55**	**92.43**	**75.65**	**78.83**	**77.29**	

RI_{calc} = Calculated retention index (HP-5MS column); RI_{lit} = literature retention index (Adams, 2009); [a]All compounds were identified by GC-MS and GC-FID according to the experiment; Sep-September; Oct–October; Nov-November; Dec-December; Jan-January. (r^2) Spearman correlations between volatile compounds from phases 1 to 5 with respect to time. Retention times (Rt) were measured in minutes without correction, and the relative percentage of each compound was determined by the signal area (2,3,55,167,168]. The RIs were calculated from the results of the analysis of a homologous series of saturated aliphatic hydrocarbons (C_8-C_{28}, Sigma-Aldrich, Brazil), performed in the same column and conditions used for analysis by GC/FID. *Non-oxygenated monoterpenes; **Oxygenated monoterpenes; ***Non-oxygenated sesquiterpenes; ****Oxygenated sesquiterpenes; ***Other compounds. For this analysis, we highlight all the constituents of the EOs of *P. mollicomum* that were observed at least once in the periods and stages of the analyzed reproductive organs.

Table S3. Weekly sums of the frequency of insect visits by potential pollinators of the inflorescences of *Piper mollicomum* Kunth from September 2020 to January 2021.

**Week**	**Insect 1** (Diptera - *Syrphidae* sp. 1)	**Insect 2** (Hymenoptera - *Tetragonisca angustula*, Latreille. 1811)	**Insect 3** (Diptera - *Syrphidae* sp. 2)	**Insect 4** (Hymenoptera - *Halictidae* sp. 1)	**Insect 5** (Hymenoptera - *Halictidae* sp. 2)	**Insect 6** (Hymenoptera - *Colletidae* sp.)
**September**						
**Day 12**	0	26	2	0	2	7
**Day 19**	0	74	3	21	5	43
**Day 30**	0	108	2	0	0	24
**October**						
**Day 14**	0	162	25	0	4	155
**Day 20**	1	371	30	2	40	168
**Day 28**	0	610	7	6	85	214
**November**						

**Day 18**	0	0	1	0	0	0
**Day 28**	0	1142	22	20	41	77
**December**						
**Day 07**	0	0	2	0	0	0
**Day 18**	0	273	59	12	52	28
**Day 25**	0	0	0	0	0	0
**January**						
**Day 02**	0	0	1	2	7	0
**Day 08**	0	2	1	4	1	2
**Day 22**	0	274	9	0	1	102

Table S4. Spearman correlation analysis between insect visitors *vs.* identified compounds in the essential oil from inflorescences of *Piper mollicomum* Kunth *vs.* microclimate from September 2020 to January 2021.

		**Spearman Correlation (r^2)**					
**Variables**		**Insect 1** *(Diptera - Syrphidae sp. 1)*	**Insect 2** *(Hymenoptera - Tetragonisca angustula Latreille. 1811)*	**Insect 3** *(Diptera - Syrphidae sp. 2)*	**Insect 4** *(Hymenoptera - Halictidae sp. 1)*	**Insect 5** *(Hymenoptera - Halictidae sp. 2)*	**Insect 6** *(Hymenoptera - Colletidae sp.)*
**Main compound**	Camphene	**1.000****	0.612	0.608	−0.441	**0.918***	**0.992****
	Limonene	0.260	−0.146	−0.226	0.395	0.011	0.266
	1,8-cineole	−0.543	0.198	−0.247	0.092	−0.348	−0.513
	Z-*β*-ocimene	−0.138	−0.395	0.636	−0.107	0.128	−0.256
	E-caryophyllene	0.645	0.806	0.186	0.356	0.606	0.648
	eupatoriochromene	0.419	0.297	**0.961****	−0.176	0.715	0.304
	α-pinene	0.593	0.581	0.846	−0.595	**0.804***	0.546
	Linalool	0.127	−0.379	−0.387	0.238	−0.187	0.155

	E-nerolidol	0.141	−0.357	0.027	0.376	0.017	0.094
	β-selinene	−0.095	−0.392	−0.480	0.532	−0.340	−0.080
	E-*β*-ocimene	0.579	0.097	0.028	0.174	0.339	0.581
	aromadendrene	**1.000****	0.612	0.608	−0.441	**0.918***	**0.992****
	Camphor	**1.000****	0.612	0.608	−0.441	**0.918***	**0.992****
	α-terpineole	−0.360	0.413	−0.233	0.091	−0.200	−0.317
	α-humulene	0.550	0.021	0.850	−0.787	0.655	0.486
	β-pinene	−0.159	0.275	0.566	−0.016	0.234	−0.239
	Myrcene	0.148	0.398	0.201	−0.648	0.238	0.188
	caryophyllene acetate	−0.402	−0.614	−0.010	−0.633	−0.364	−0.396
	δ-terpineole	−0.250	−0.381	−0.454	−0.605	−0.435	−0.159
	β-elemene	−0.250	−0.381	−0.454	−0.605	−0.435	−0.159
	benzyl benzoate	−0.250	−0.384	0.587	−0.114	0.050	−0.362
	γ-terpinene	−0.250	−0.384	0.587	−0.114	0.050	−0.362
	tolualdehyde	−0.250	−0.384	0.587	−0.114	0.050	−0.362
	germacren D	−0.250	−0.381	−0.454	−0.605	−0.435	−0.159
	δ-cadinene	−0.262	−0.402	0.575	−0.139	0.033	−0.371
**Abiotic factors**	Wind speed (m/s)	−0.174	0.284	0.413	0.491	0.291	0.176
	Relative humidity (%)	−0.311	**−0.667***	−0.394	−0.319	−0.512	**−0.623***
	Local temperature (°C)	0.034	**0.749***	**0.633***	0.303	**0.606***	**0.642***
	Inflorescence temperature (°C)	0.104	**0.688***	**0.559***	0.340	**0.606***	**0.616***
	Leaf temperature (°C)	0.173	**0.763***	**0.626***	0.437	**0.717***	**0.692***
	Rain (mm)	−0.144	**−0.649***	**−0.657***	−0.245	−0.279	**−0.649***
	Radiation (kJ/m²)	0.241	**0.869***	0.530	0.327	0.443	0.749
	Light intensity (kJ/m²)	0.104	0.497	0.296	**0.538***	**0.560***	0.281

$^{*}p < 0.05$; $^{**}p < 0.01$.

Table S5. Spearman´s correlation analysis between microclimate *vs.* identified compounds in the essential oil from inflorescences of *Piper mollicomum* Kunth from September 2020 to January 2021.

Compounds	Spearman Correlation (r^2)						
	Wind Speed (m/s)	Relative Humidity (%)	Temperature (°C)			Rain (mm)	Light Intensity (kJ/m²)
			Local Averages	Inflor.	Leaf		
**camphene**	**1.000****	**0.067**	**0.784**	−0.045	−0.122	−0.256	−0.408
limonene	0.260	−0.299	−0.187	0.540	0.484	0.867	0.375
1,8-cineole	−0.543	−0.093	0.074	−0.364	−0.291	−0.548	−0.406
Z-*β*-ocimene	−0.138	**0.949***	−0.430	−0.597	−0.597	−0.124	0.711
E-caryophyllene	0.645	−0.208	0.657	−0.113	−0.173	0.121	−0.330
eupatoriochromene	0.419	**0.911***	0.246	−0.833	−0.869	−0.435	0.241
α-pinene	0.593	0.538	0.699	−0.661	−0.684	**−0.880***	−0.428
linalool	0.127	−0.409	−0.331	0.749	0.708	**0.882***	0.372
E-nerolidol	0.141	0.126	−0.426	0.271	0.220	0.828	0.721
β-selinene	−0.095	−0.367	−0.478	0.618	0.590	**0.985****	0.512
E-*β*-ocimene	0.579	−0.229	0.124	0.439	0.365	0.640	0.169
aromadendrene	**1.000****	0.067	0.784	−0.045	−0.122	−0.256	−0.408
camphor	**1.000****	0.067	0.784	−0.045	−0.122	−0.256	−0.408
α-terpineole	−0.360	−0.215	0.293	−0.334	−0.274	−0.580	−0.589
α-humulene	0.550	0.672	0.277	−0.354	−0.388	−0.560	0.012
β-pinene	−0.159	0.717	0.123	**−0.945***	**−0.916***	−0.682	0.021
myrcene	0.148	−0.033	0.581	−0.245	−0.216	**−0.927***	−0.775
caryophyllene acetate	−0.402	0.218	−0.370	0.065	0.116	−0.395	0.069
δ-terpineole	−0.250	−0.468	−0.078	0.564	0.609	−0.256	−0.408
β-elemene	−0.250	−0.468	−0.078	0.564	0.609	−0.256	−0.408
benzyl benzoate	−0.250	**0.935***	−0.431	−0.654	−0.639	−0.233	0.640
γ-terpinene	−0.250	**0.935***	−0.431	−0.654	−0.639	−0.233	0.640
tolualdehyde	−0.250	**0.935***	−0.431	−0.654	−0.639	−0.233	0.640
germacren D	−0.250	−0.468	−0.078	0.564	0.609	−0.256	−0.408
δ-cadinene	−0.262	**0.926***	−0.438	−0.638	−0.621	−0.245	0.630

*$p < 0.05$; ** $p < 0.01$; Inflor. – Inflorescence.

Table S6. Spearman´s correlation analysis between insect visitors *vs.* pattern of phenological events of *Piper mollicomum* Kunth from September 2020 to January 2021.

Variables analyzed		Spearman Correlation (r^2)					
		**Insect 1** (Diptera - *Syrphidae* sp. 1)	**Insect 2** (Hymenoptera - *Tetragonisca angustula* Latreille. 1811)	**Insect 3** (Diptera - *Syrphidae* sp. 2)	**Insect 4** (Hymenoptera - *Halictidae* sp. 1)	**Insect 5** (Hymenoptera - *Halictidae* sp. 2)	**Insect 6** (Hymenoptera - *Colletidae* sp.)
**Phenophases**	Leaf Budding	0.000	−0.161	−0.025	−0.271	−0.341	0.007
	Leaf Fall	−0.144	−0.009	0.176	−0.088	0.059	0.213
	Mature Inflorescence	0.172	0.236	0.284	0.101	0.169	0.142
	Immature Inflorescence	0.311	0.276	0.278	0.019	0.155	0.332
	Number of Inflorescence	0.173	0.242	0.317	0.087	0.141	0.168
	Immature Infrutescence	−0.318	−0.306	−0.399	−0.002	−0.165	−0.447
	Mature Infrutescence	−0.077	0.243	0.173	−0.257	−0.139	0.243

Table S7. Spearman´s correlation analysis between abiotic factors *vs.* pattern of phenological events of *Piper mollicomum* Kunth from September 2020 to January 2021.

Variables analyzed	Spearman Correlation (r^2)				
	**Temperature (°C)**	**Humidity (%)**	**Wind (m/s)**	**Rain (mm)**	**Light Intensity (kJ/m²)**
Leaf Budding	−0.600	0.600	0.000	**0.800***	**−0.800***
Leaf Fall	**−0.783***	0.224	0.335	0.335	−0.335
Mature Inflorescence	0.100	−0.100	0.500	−0.300	0.300
Immature Inflorescence	−0.400	0.000	0.600	−0.200	0.200

Number of Inflorescence	0.100	−0.100	0.500	−0.300	0.300
Immature Infrutescence	0.600	−0.100	−0.600	−0.200	0.200
Mature Infrutescence	0.354	−0.354	−0.354	−0.354	0.354

* $p < 0.05$.

Table S8. Characterization of the collection sites of *Piper mollicomum* Kunth accessions from the Tijuca National Park (PM-TNP), city of Rio de Janeiro, RJ.

Site	Latitude (S)/ Longitude (W)	Elevation (m)	Voucher number
PM-TNP1	22°58'08.2" / 43°14'24.1"	127	HRJ13444
PM-TNP2	22°58'09.0"/ 43°14'26.3"	71	HRJ13445
PM-TNP3	22°58'14.0"/ 43°14'32.9"	92	HRJ13448
PM-TNP4	22°58'17.8"/ 43°14'31.9"	68	HRJ13449
PM-TNP5	22°58'20.2"/43°14'33.6"	74	HRJ13447
PM-TNP6	22°58'21.3"/ 43°14'34.2"	87	HRJ13446

Table S9. Constituents of essential oils from leaves and different stages of inflorescences of *Piper mollicomum* Kunth.

***Piper mollicomum* - September/2020**							
Compounds	tR	IRKc	IRKlit	% in 1	IM	Average	SD
Leaf							
3E-Hexenol	3.793	845	844	0.39	ADAMS	0.669	0.008
α-pinene	5.395	932	932	2.13	ADAMS	2.385	0.007
camphene	5.816	934	946	0.87	ADAMS	0.923	0.004
β-pinene	6.578	941	974	5.43	ADAMS	6.304	0.024
myrcene	6.844	943	988	0.47	ADAMS	0.476	0.009

limonene	8.142	1032	1024	2.26	ADAMS	3.763	0.016
1,8-cineole	8.233	1032	1026	1.91	ADAMS	0.868	0.004
linalool	10.924	1088	1008	37.16	ADAMS	44.849	0.114
α-fenchocamphorone	10.988	1088	1104	0.62	ADAMS	0.321	0.018
camphor	12.534	1142	1141	1.78	NIST	1.574	0.003
borneol	13.527	1147	1165	0.11	NIST/ADAMS	0.085	0.002
α-terpineol	14.519	1150	1186	0.43	ADAMS	0.391	0.005
isopulegyl acetate	18.083	1285	1283	0.41	ADAMS	0.204	0.078
bornyl acetate	18.21	1286	1284	0.22	ADAMS	0.069	0.003
undecanone	18.653	1290	1293	0.76	ADAMS	0.730	0.006
undecanol	19.104	1305	1301	0.30	ADAMS	0.041	0.002
δ-elemene	20.199	1346	1335	0.36	ADAMS	0.084	0.001
β-elemene	22.637	1376	1389	3.13	ADAMS	2.456	0.006
α-gurjunene	23.291	1410	1409	0.10	ADAMS	0.104	0.001
E-caryophyllene	23.796	1415	1417	2.07	ADAMS	2.331	0.004
γ-elemene	24.247	1418	1434	1.12	ADAMS	1.651	0.004
α-humulene	25.247	1455	1452	1.66	ADAMS	1.332	0.003
aromadendrene	25.417	1457	1439	0.22	ADAMS	0.278	0.001
α-amorphene	26.084	1462	1483	0.23	ADAMS	0.213	0.001
germacrene D	26.322	1463	1480	4.45	ADAMS	2.344	0.010
cis-4,10-epoxy-amorphane	26.585	1464	1481	0.76	ADAMS	0.224	0.070
α-cubebene	26.705	1491	1348	0.20	ADAMS	0.210	0.002
bicyclogermacrene	26.879	1492	1500	2.33	ADAMS	1.531	0.696
tridecanone	27.028	1471	1495	0.50	ADAMS	0.315	0.011
E,E-α-farnesene	27.348	1507	1505	0.39	ADAMS	0.258	0.048
γ-cadinene	27.579	1511	1513	0.19	ADAMS	0.166	0.015
δ-cadinene	27.807	1511	1522	0.90	ADAMS	1.000	0.109
β-panasinsene	28.476	1510	1381	0.16	ADAMS	0.133	0.020
cis-muurola-3,5-diene	28.664	1512	1448	0.14	ADAMS	0.274	0.027
elemol acetate	28.983	1542	1559	0.36	ADAMS	0.163	0.026
germacrene B	29.292	1544	1561	0.50	ADAMS	0.146	0.001
E-nerolidol	29.579	1546	1556	2.75	ADAMS	2.174	0.013

maaliol	29.771	1548	1566	0.18	ADAMS	0.129	0.026
caryophyllene oxide	29.955	1591	1496	0.11	ADAMS	0.137	0.037
guaiol	30.043	1592	1600	0.33	ADAMS	0.242	0.082
spathulenol	30.383	1587	1577	0.25	ADAMS	0.166	0.048
valencene	31.096	1591	1496	1.56	ADAMS	0.553	0.006
α-copaene	31.51	1627	1374	0.15	ADAMS	0.066	0.042
α-selinene	31.572	1627	1498	0.15	ADAMS	0.075	0.044
aromadendrene epoxide	31.811	1628	1639	5.98	ADAMS	1.216	0.054
cis-cadina-1,4-diene	31.989	1628	1495	1.06	ADAMS	0.530	0.038
10-epi-γ-eudesmol	32.184	1631	1638	0.46	ADAMS	0.457	0.023
α-muurolol	32.541	1632	1640	1.24	ADAMS	4.270	0.025
pogostol	33.104	1638	1651	0.47	ADAMS	1.419	0.083
selin-11-en-4-α-ol	33.484	1640	1652	0.22	ADAMS	0.364	0.018
α-himachal-4-en-1-β-ol	33.645	1642	1699	2.17	ADAMS	1.506	0.014
eudesm-7(11)-en-4-ol	34.537	1696	1700	0.46	ADAMS	0.354	0.003
benzyl benzoate	37.023	1765	1759	0.61	ADAMS	0.774	0.016
**Overall**				**97.62**		**97.835**	
Inflorescence Stage 1							
camphene	11.224	937	946	12.72	ADAMS	25.410	0.206
limonene	11.508	1038	1024	41.81	ADAMS	8.394	0.323
1,8-cineole	11.605	1033	1026	5.45	ADAMS	8.222	0.291
Z-β-ocimene	12.744	1034	1032	10.90	ADAMS	3.293	0.172
E-caryophyllene	27.047	1415	1417	5.45	ADAMS	5.363	0.072
eupatoriochromene	36.632	1639	1761	23.63	ADAMS	8.546	0.115
**Overall**				**99.96**		**59.229**	
Inflorescence Stage 2							
α-pinene	6.725	936	932	15.68	ADAMS	18.220	0.081
limonene	8.235	1038	1024	21.56	ADAMS	9.175	0.067
linalool	10.727	1088	1008	35.29	ADAMS	29.239	0.029
E-caryophyllene	23.79	1415	1417	2.94	ADAMS	4.417	0.016
E-nerolidol	29.569	1546	1556	2.94	ADAMS	2.278	0.020
eupatoriochromene	33.636	1639	1761	21.56	ADAMS	6.345	0.012

**Overall**				**99.97**		**69.673**	
Inflorescence Stage 3							
limonene	8.307	1039	1024	12.05	ADAMS	14.578	0.849
Z-β-ocimene	8.424	1035	1032	0.85	ADAMS	0.732	0.139
E-β-ocimene	8.79	1037	1044	2.20	ADAMS	2.243	0.105
γ-terpinene	9.217	1037	1054	0.07	ADAMS	0.113	0.027
terpinolene	10.181	1084	1086	0.28	ADAMS	0.325	0.002
linalool	11.085	1088	1008	47.08	ADAMS	62.023	2.755
allo-ocimene	11.875	1096	1128	0.07	ADAMS	0.206	0.120
camphor	12.587	1142	1141	0.24	NIST	0.969	0.014
E-caryophyllene	23.834	1416	1417	3.62	ADAMS	2.123	1.511
β-selinene	25.276	1464	1489	2.66	ADAMS	2.391	0.265
E-nerolidol	29.598	1546	1556	3.24	ADAMS	2.041	0.487
eupatoriochromene	33.759	1639	1761	11.95	ADAMS	5.911	0.027
**Overall**				**84.30**		**93.655**	
Inflorescence Stage 4							
limonene	8.297	1039	1024	6.82	ADAMS	6.299	0.028
Z-β-ocimene	8.43	1035	1032	1.50	ADAMS	0.814	0.003
E-β-ocimene	8.821	1037	1044	3.78	ADAMS	3.205	0.014
cis-linalool oxide	9.66	1047	1067	0.74	ADAMS	0.150	0.002
terpinolene	10.18	1084	1086	0.10	ADAMS	0.192	0.002
linalool	11.222	1088	1008	54.10	ADAMS	73.136	0.061
allo-ocimene	11.917	1096	1128	0.15	ADAMS	0.041	0.000
γ-terpinene	13.989	1149	1054	0.08	ADAMS	0.058	0.002
α-terpineol	14.595	1150	1186	0.89	ADAMS	0.022	0.006
α-copaene	22.033	1365	1374	0.08	ADAMS	0.050	0.000
β-elemene	22.632	1376	1389	0.13	ADAMS	0.084	0.002
E-caryophyllene	23.864	1416	1417	3.96	ADAMS	2.480	0.003
germacrene D	24.246	1426	1480	0.08	ADAMS	0.033	0.000
β-selinene	25.3	1464	1489	2.94	ADAMS	1.718	0.000
α-selinene	26.888	1508	1498	0.30	ADAMS	0.136	0.004
α-muurolene	27.036	1510	1500	0.10	ADAMS	0.059	0.006

E,E-α-farnesene	27.376	1513	1505	1.42	ADAMS	0.908	0.003
δ-cadinene	27.815	1517	1522	0.23	ADAMS	0.112	0.001
epizonarene	27.968	1518	1501	0.10	ADAMS	0.008	0.001
elemol acetate	28.76	1515	1545	0.05	ADAMS	0.063	0.002
E-nerolidol	29.63	1546	1556	3.80	ADAMS	2.249	0.005
caryophyllene oxide	30.217	1593	1496	0.25	ADAMS	0.193	0.000
valencene	31.1	1591	1496	0.20	ADAMS	0.124	0.001
α-selinene	31.586	1626	1498	0.10	ADAMS	0.192	0.001
aromadendrene epoxide	31.769	1628	1639	1.19	ADAMS	1.057	0.002
eupatoriochromene	33.813	1639	1761	12.30	ADAMS	4.837	0.009
benzyl benzoate	37.035	1765	1759	0.23	ADAMS	0.081	0.000
**Overall**				**96.15**		**98.649**	

***Piper mollicomum* - Octuber/2020**							
Compounds	tR	IRKc	IRKlit	% IN1	IM	Average	SD.
Folha							
3E-Hexenol	3.801	845	844	0.33	ADAMS	0.609	0.008
α-pinene	5.394	932	932	1.70	ADAMS	1.804	0.001
camphene	5.82	934	946	3.48	ADAMS	3.841	0.001
β-pinene	6.563	941	974	2.13	ADAMS	2.370	0.002
myrcene	6.845	943	988	0.56	ADAMS	0.626	0.001
limonene	8.149	1032	1024	4.79	ADAMS	5.405	0.005
E-β-ocimene	8.706	1037	1044	0.27	ADAMS	0.281	0.002
cis-linalool oxide	9.584	1047	1067	0.19	ADAMS	0.222	0.001
linalool	10.821	1087	1088	22.33	ADAMS	27.205	0.073
α-fenchocamphorone	10.921	1088	1104	0.16	ADAMS	0.082	0.001
camphor	12.544	1142	1141	4.45	NIST	4.498	0.009
borneol	13.52	1147	1165	0.56	NIST/ADAMS	0.550	0.003
α-terpineol	14.525	1150	1186	0.29	ADAMS	0.309	0.002
bornyl acetate	18.208	1286	1284	0.28	ADAMS	0.093	0.000

undecanone	18.663	1290	1293	0.41	ADAMS	0.032	0.000
δ-elemene	20.345	1347	1014	0.28	ADAMS	0.397	0.001
α-copaene	22.013	1365	1374	0.23	ADAMS	0.200	0.000
β-elemene	22.614	1376	1389	3.80	ADAMS	1.688	0.006
α-gurjunene	23.292	1410	1409	0.25	ADAMS	0.247	0.001
E-caryophyllene	23.814	1415	1417	4.40	ADAMS	3.542	0.002
γ-elemene	24.247	1418	1434	1.45	ADAMS	4.875	0.008
β-selinene	25.262	1455	1452	3.26	ADAMS	2.329	0.004
aromadendrene	25.422	1457	1439	0.60	ADAMS	0.111	0.000
δ-selinene	25.982	1462	1492	0.32	ADAMS	0.258	0.001
α-amorphene	26.088	1462	1483	0.30	ADAMS	0.333	0.000
germacrene D	26.335	1463	1480	6.25	ADAMS	2.884	0.001
cis-4,10-epoxy-amorphane	26.596	1464	1481	0.75	ADAMS	0.588	0.003
α-cubebene	26.712	1491	1348	0.30	ADAMS	0.123	0.000
bicyclogermacrene	26.892	1492	1500	3.96	ADAMS	3.274	0.021
α-muurolene	27.034	1494	1500	0.60	ADAMS	0.482	0.000
E,E-α-farnesene	27.376	1507	1505	3.64	ADAMS	2.272	0.143
δ-cadinene	27.818	1511	1522	1.40	ADAMS	2.814	0.008
epizonarene	27.967	1512	1501	0.29	ADAMS	0.387	0.001
E-nerolidol	29.599	1546	1556	5.10	ADAMS	1.270	0.003
guaiol	30.045	1592	1600	0.31	ADAMS	0.243	0.001
caryophyllene oxide	30.208	1594	1496	0.37	ADAMS	0.070	0.001
spathulenol	30.391	1587	1577	0.61	ADAMS	0.089	0.001
valencene	31.095	1591	1496	1.60	ADAMS	0.313	0.021
α-copaene	31.507	1627	1374	0.31	ADAMS	0.163	0.003
aromadendrene epoxide	31.785	1628	1639	4.85	ADAMS	4.536	0.009
cis-cadina-1,4-diene	31.981	1628	1495	0.68	ADAMS	0.363	0.004
10-epi-γ-eudesmol	32.196	1631	1638	0.18	ADAMS	0.062	0.000
α-muurolol	32.536	1632	1640	1.83	ADAMS	1.293	0.001
Z-bisaboladien-4-ol	33.636	1644	1618	2.25	ADAMS	3.737	0.167
benzyl benzoate	37.023	1765	1759	1.34	ADAMS	0.587	0.001

sclareolide	48.189	2050	2065	0.26	ADAMS	0.240	0.005
**Overall**				**97.08**		**88.809**	
Inflorescence stage 1							
limonene	8.262	1038	1024	13.99	ADAMS	17.851	0.265
linalool	10.854	1087	1088	35.32	ADAMS	44.290	0.080
camphor	12.555	1142	1141	1.73	NIST	2.008	0.008
borneol	13.556	1147	1165	0.60	NIST/ADAMS	0.685	0.000
α-terpineol	14.546	1150	1186	1.30	ADAMS	1.486	0.004
E-caryophyllene	23.793	1415	1417	1.99	ADAMS	2.675	0.028
α-humulene	25.243	1455	1452	1.47	ADAMS	0.225	0.003
E,E-α-farnesene	27.354	1507	1505	0.69	ADAMS	0.132	0.002
E-nerolidol	29.599	1546	1556	2.33	ADAMS	2.901	0.079
eupatoriochromene	33.724	1639	1761	26.25	ADAMS	16.126	0.114
**Overall**				**85.66**		**88.380**	
Inflorescence stage 2							
limonene	8.137	1038	1024	21.44	ADAMS	20.074	4.169
linalool	10.695	1088	1008	36.53	ADAMS	46.208	8.756
E-caryophyllene	23.785	1415	1417	3.54	ADAMS	2.756	0.211
α-humulene	25.239	1455	1452	2.42	ADAMS	2.423	0.421
eupatoriochromene	33.636	1639	1761	20.13	ADAMS	14.988	2.529
**Overall**				**84.06**		**86.450**	
Inflorescence stage 3							
α-pinene	5.694	936	932	4.29	ADAMS	4.29	
camphene	6.062	934	946	7.17	ADAMS	7.17	
β-pinene	6.73	943	974	3.16	ADAMS	3.16	
myrcene	6.974	945	988	1.13	ADAMS	1.13	
limonene	8.273	1039	1024	7.44	ADAMS	7.44	
1,8-cineole	8.336	1033	1026	0.16	ADAMS	0.16	
Z-β-ocimene	8.416	1035	1032	0.74	ADAMS	0.74	
E-β-ocimene	8.784	1037	1044	2.10	ADAMS	2.10	

cis-linalool oxide	9.648	1047	1067	0.39	ADAMS	0.39	
terpinolene	10.179	1084	1086	0.16	ADAMS	0.16	
linalool	10.963	1088	1008	30.27	ADAMS	30.27	
camphor	12.672	1142	1141	11.45	NIST	11.45	
camphene hydrate	12.898	1144	1145	0.16	ADAMS	0.16	
borneol	13.615	1147	1165	3.39	NIST/ADAMS	3.39	
trans-β-terpineol	13.957	1149	1159	0.12	ADAMS	0.12	
α-terpineol	14.556	1150	1186	0.86	ADAMS	0.86	
bornyl acetate	18.229	1286	1284	0.39	ADAMS	0.39	
β-elemene	22.623	1376	1389	0.16	ADAMS	0.16	
E-caryophyllene	23.825	1416	1417	3.47	ADAMS	3.47	
α-humulene	25.268	1455	1452	2.49	ADAMS	2.49	
aromadendrene	25.435	1457	1439	0.16	ADAMS	0.16	
germacrene D	26.29	1426	1480	0.47	ADAMS	0.47	
β-selinene	26.596	1464	1489	0.16	ADAMS	0.16	
α-selinene	26.881	1508	1498	0.35	ADAMS	0.35	
α-muurolene	27.031	1510	1500	0.19	ADAMS	0.19	
E,E-α-farnesene	27.352	1513	1505	0.78	ADAMS	0.78	
δ-cadinene	27.807	1517	1522	0.35	ADAMS	0.35	
E-nerolidol	29.587	1546	1556	2.92	ADAMS	2.92	
caryophyllene oxide	30.208	1593	1496	0.19	ADAMS	0.19	
cis-β-guaiene	31.088	1620	1492	0.31	ADAMS	0.31	
Z-α-bisabolene	31.271	1622	1506	0.27	ADAMS	0.27	
aromadendrene epoxide	31.749	1628	1639	0.86	ADAMS	0.86	
cis-cadina-1,4-diene	31.981	1628	1495	0.27	ADAMS	0.27	
α-muurolol	32.528	1632	1640	0.31	ADAMS	0.31	
α-copaene	32.698	1633	1640	0.23	ADAMS	0.23	
eupatoriochromene	33.754	1639	1761	13.21	ADAMS	13.21	
**Overall**				**101.17**		**101.17**	
Inflorescence stage 4							

camphene	5.808	934	946	7.40	ADAMS	6.377	0.006
limonene	8.135	1039	1024	5.71	ADAMS	3.118	0.003
linalool	10.685	1088	1008	54.55	ADAMS	51.961	0.024
camphor	12.506	1142	1141	12.47	NIST	11.265	0.007
borneol	13.522	1147	1165	6.10	NIST	4.917	0.003
E-caryophyllene	23.786	1416	1417	2.08	ADAMS	3.371	0.029
eupatoriochromene	33.646	1639	1761	9.87	ADAMS	5.632	1.254
**Overall**				**98.18**		**86.641**	

***Piper mollicomum* - november/2020**							
Compounds	tR	IRKc	IRKlit	% IN1	IM	Average	SD.
Folha							
α-pinene	6.654	928	932	11.33	ADAMS	1.362	0.009
β-pinene	7.937	941	974	9.97	ADAMS	5.027	0.044
1,8-cineole	9.941	1037	1026	14.75	ADAMS	2.701	0.020
γ-terpinene	10.622	1045	1054	0.70	ADAMS	0.115	0.013
Z-β-ocimene	11.58	1033	1032	0.17	ADAMS	0.113	0.005
terpinolene	11.636	1071	1086	0.29	ADAMS	0.115	0.001
linalool	12.149	1087	1088	0.76	ADAMS	0.338	0.053
ocimene	13.267	1119	1128	0.31	ADAMS	0.328	0.017
α-terpineol	16.264	1286	1284	2.81	ADAMS	11.418	0.035
undecanol	20.632	1309	1301	0.25	ADAMS	0.143	0.008
linalool propanoate	21.479	1322	1334	2.22	ADAMS	9.198	0.008
δ-elemene	22.054	1346	1335	0.58	ADAMS	1.857	0.003
β-elemene	24.063	1376	1389	5.28	ADAMS	4.453	0.035
α-gurjunene	25.076	1410	1409	0.11	ADAMS	0.561	0.002
β-gurjunene	25.796	1415	1433	5.73	ADAMS	5.624	0.013
E-caryophyllene	25.842	1415	1417	0.55	ADAMS	0.833	0.021
γ-elemene	26.201	1418	1434	4.04	ADAMS	15.950	0.064
aromadendrene	26.463	1457	1439	0.13	ADAMS	0.465	0.013

cis-cadina-1(6),4-diene	26.696	1455	1461	1.15	ADAMS	4.392	0.007
cis-muurola-4(14),5-diene	27	1457	1465	1.57	ADAMS	1.025	0.005
linalool isovalerate	27.235	1461	1466	2.61	ADAMS	2.527	0.004
trans-muurola-4(14),5-diene	27.945	1492	1493	1.07	ADAMS	1.539	0.014
germacrene D	28.248	1495	1480	2.93	ADAMS	4.434	0.023
β-selinene	28.483	1498	1452	0.80	ADAMS	0.887	0.008
γ-cadinene	28.581	1511	1513	0.75	ADAMS	0.717	0.004
germacrene B	28.804	1516	1559	1.63	ADAMS	1.143	0.012
E,E-α-farnesene	29.098	1524	1505	1.55	ADAMS	1.364	0.013
δ-cadinene	29.675	1529	1522	1.53	ADAMS	1.295	0.014
E-nerolidol	31.328	1546	1561	2.09	ADAMS	2.096	0.016
α-cadinol	34.921	1793	1652	0.82	ADAMS	0.982	0.002
intermedeol	35.007	1794	1658	0.49	ADAMS	0.146	0.002
eupatoriochromene	35.477	1798	1761	1.87	ADAMS	2.567	0.015
caryophyllene acetate	36.769	1817	1701	1.21	ADAMS	0.313	0.003
benzyl benzoate	38.805	1765	1759	0.70	ADAMS	0.272	0.004
aromadendrene epoxide	31.785	1628	1639	4.85	ADAMS	1.645	0.008
cis-cadina-1,4-diene	31.981	1628	1495	0.68	ADAMS	0.156	0.003
α-muurolol	32.536	1632	1640	1.83	ADAMS	0.852	0.026
α-copaene	32.704	1633	1640	0.52	ADAMS	0.018	0.002
Z-bisaboladien-4-ol	33.636	1644	1618	2.25	ADAMS	0.043	0.001
benzyl benzoate	37.023	1765	1759	1.34	ADAMS	0.177	0.014
sclareolide	48.189	2050	2065	0.26	ADAMS	0.026	0.004
**Overall**				**96.38**		**89.254**	
Inflorescence stage 1							
α-pinene	8.93	937	932	1.97	ADAMS	10.545	1.016
β-pinene	9.502	941	974	4.56	ADAMS	17.129	0.356
myrcene	9.561	943	988	1.49	ADAMS	1.672	0.013
α-phellandrene	9.963	979	1002	0.14	ADAMS	0.424	0.055
α-terpinene	10.205	1000	1014	0.42	ADAMS	1.154	0.116
1,8-cineole	10.81	996	1026	17.54	ADAMS	26.078	8.836

2,3-diethyl-pyrazine	13.397	1088	1081	0.18	ADAMS	0.065	0.015
α-terpineol	15.17	1150	1162	7.68	ADAMS	2.308	0.143
E-nerolidol	31.147	1546	1561	1.02	ADAMS	0.200	0.025
germacrene D-4-ol	33.85	1567	1574	0.17	ADAMS	0.088	0.009
eupatoriochromene	35.784	1798	1761	33.36	ADAMS	11.848	0.598
caryophyllene acetate	36.769	1817	1701	1.18	ADAMS	2.066	0.473
**Overall**				**69.71**		**73.577**	
Inflorescence stage 2							
α-pinene	8.869	937	932	3.02	ADAMS	9.347	1.326
sabinene	9.3	954	969	1.12	ADAMS	3.072	0.290
β-pinene	9.546	942	932	6.99	ADAMS	8.151	0.377
myrcene	9.623	943	988	1.46	ADAMS	1.245	0.067
α-phellandrene	9.948	979	1002	0.15	ADAMS	0.217	0.024
α-terpinene	10.185	1000	1014	0.61	ADAMS	0.698	0.030
1,8-cineole	10.878	1035	1026	22.83	ADAMS	44.917	0.942
linalool	12.758	1088	1098	4.67	ADAMS	4.146	0.177
trans-sabinene hydrate	12.821	1090	1098	0.17	ADAMS	0.118	0.005
ocimene	13.469	1120	1128	0.66	ADAMS	0.206	0.005
camphor	14.295	1142	1141	0.10	ADAMS	0.018	0.000
α-terpineol	15.31	1177	1186	11.63	ADAMS	3.392	0.347
pinocamphone	15.726	1245	1247	1.40	ADAMS	0.011	0.001
α-copaene	23.748	1365	1374	0.10	ADAMS	0.690	0.051
β-elemene	24.322	1376	1389	0.28	ADAMS	0.090	0.001
E-caryophyllene	25.664	1415	1417	1.66	ADAMS	0.049	0.013
germacrene D	25.972	1495	1480	0.32	ADAMS	0.086	0.004
γ-himachalene	26.864	1479	1481	0.14	ADAMS	0.073	0.002
α-humulene	27.109	1480	1452	1.26	ADAMS	0.013	0.002
β-selinene	28.362	1498	1452	0.14	ADAMS	0.023	#DIV/0!
α-selinene	28.654	1500	1498	0.17	ADAMS	0.012	0.000
δ-cadinene	29.547	1529	1522	0.31	ADAMS	0.012	0.001
E-nerolidol	31.243	1546	1561	0.96	ADAMS	0.041	0.003

caryophyllene oxide	32.003	1594	1582	0.11	ADAMS	0.113	0.006
epi-α-cadinol	34.273	1789	1638	0.26	ADAMS	0.189	0.017
eupatoriochromene	36.149	1653	1761	23.74	ADAMS	8.408	0.594
cis-thujopsenal	36.927	1707	1708	0.74	ADAMS	0.120	0.152
benzyl benzoate	38.793	1765	1759	0.20	ADAMS	0.033	0.001
**Overall**				**85.20**		**85.479**	
Inflorescence stage 3							
α-pinene	8.827	936	932	2.87	ADAMS	2.768	2.215
β-pinene	9.555	943	974	5.24	ADAMS	6.802	0.119
myrcene	9.629	945	988	1.38	ADAMS	1.269	0.044
α-phellandrene	9.939	979	1002	0.11	ADAMS	0.206	0.066
α-terpinene	10.178	1000	1014	0.48	ADAMS	0.289	0.009
1,8-cineole	10.961	1033	1026	22.01	ADAMS	51.276	0.320
γ-terpinene	11.404	1045	1054	1.43	ADAMS	0.625	0.019
tolualdehyde	11.869	1054	1062	0.42	ADAMS	0.016	0.001
terpinolene	12.271	1071	1086	0.47	ADAMS	0.789	0.039
linalool	12.938	1088	1008	4.37	ADAMS	2.480	0.054
trans-sabinene hydrate	12.994	1090	1098	0.18	ADAMS	0.026	0.000
ocimene	13.592	1120	1128	0.90	ADAMS	0.063	0.002
α-terpineol	15.064	1150	1162	1.94	ADAMS	0.750	0.012
terpinen-4-ol	15.876	1189	1174	0.90	ADAMS	0.518	0.007
bornyl acetate	16.357	1286	1284	9.43	ADAMS	5.612	0.096
δ-elemene	22.058	1346	1335	0.21	ADAMS	0.012	0.001
α-copaene	23.777	1365	1374	0.21	ADAMS	0.086	0.059
β-elemene	24.413	1376	1389	0.75	ADAMS	0.034	0.007
E-caryophyllene	25.734	1416	1417	2.91	ADAMS	1.439	0.052
germacrene D	26.054	1426	1480	1.22	ADAMS	0.262	0.011
trans-cadina-1(6),4-diene	26.609	1465	1475	0.22	ADAMS	0.086	0.010
9-epi-E-caryophyllene	27.248	1460	1464	2.16	ADAMS	1.334	0.026
γ-curcumene	27.945	1480	1481	0.15	ADAMS	0.130	0.013
β-selinene	28.43	1498	1452	0.25	ADAMS	0.074	0.002

γ-cadinene	28.537	1511	1513	0.15	ADAMS	0.266	0.003
α-selinene	28.723	1500	1498	0.38	ADAMS	0.111	0.004
E,E-α-farnesene	29.051	1524	1505	0.63	ADAMS	0.207	0.003
δ-cadinene	29.61	1529	1522	0.30	ADAMS	0.226	0.002
E-nerolidol	31.308	1546	1561	1.16	ADAMS	0.259	0.015
caryophyllene oxide	32.049	1593	1496	0.19	ADAMS	0.035	0.005
2E,4E-dodecadienal	33.933	1780	1518	0.30	ADAMS	0.070	0.001
epi-α-cadinol	34.308	1789	1638	0.26	ADAMS	0.122	0.002
eupatoriochromene	36.372	1639	1761	24.25	ADAMS	9.009	0.179
cis-thujopsenal	37.096	1707	1708	0.87	ADAMS	1.008	0.238
benzyl benzoate	38.842	1765	1759	0.15	ADAMS	0.037	0.008
**Overall**				**88.85**		**88.297**	
Inflorescence stage 4							
α-pinene	8.503	936	932	2.40	ADAMS	6.803	1.370
β-pinene	9.566	943	974	9.87	ADAMS	5.397	0.040
1,8-cineole	10.793	1036	1026	16.19	ADAMS	58.780	1.379
linalool	13.549	1091	1098	8.01	ADAMS	4.066	0.152
ocimene	14.152	1119	1128	1.53	ADAMS	0.281	0.006
α-terpineol	14.642	1150	1162	0.49	ADAMS	0.013	0.001
camphor	14.828	1152	1141	0.30	ADAMS	0.018	0.001
δ-terpineol	15.624	1160	1162	1.68	ADAMS	0.311	0.031
bornyl acetate	16.425	1286	1284	3.46	ADAMS	0.611	0.013
α-terpineol	17.588	1287	1186	11.33	ADAMS	6.059	0.220
Geraniol	17.917	1288	1249	0.24	ADAMS	0.008	0.003
neryl acetate	23.858	1350	1359	0.40	ADAMS	0.029	0.001
β-elemene	24.403	1376	1389	0.24	ADAMS	0.085	0.114
E-caryophyllene	25.861	1415	1417	3.88	ADAMS	0.606	0.143
β-gurjunene	26.103	1430	1431	0.36	ADAMS	0.031	0.001
α-humulene	27.34	1551	1452	3.89	ADAMS	0.357	0.018
γ-curcumene	27.969	1480	1481	1.20	ADAMS	0.448	0.005
germacrene D	28.134	1482	1480	0.28	ADAMS	0.045	0.011

β-selinene	28.46	1498	1489	0.32	ADAMS	0.029	0.010
cis-cadina-1,4-diene	28.563	1499	1495	0.37	ADAMS	0.075	0.003
α-selinene	28.751	1500	1498	0.48	ADAMS	0.094	0.005
E,E-α-farnesene	29.112	1524	1505	1.37	ADAMS	0.053	0.049
γ-cadinene	29.644	1525	1513	0.53	ADAMS	0.061	0.001
E-nerolidol	31.423	1546	1561	1.63	ADAMS	0.206	0.018
caryophyllene oxide	32.121	1593	1496	0.34	ADAMS	0.019	0.001
zerumbone	33.186	1728	1732	0.34	ADAMS	0.039	0.001
α-copaene	34.528	1757	1374	0.19	ADAMS	0.013	0.002
eupatoriochromene	36.299	1801	1761	20.04	ADAMS	6.836	0.207
caryophyllene acetate	36.769	1817	1701	1.02	ADAMS	0.350	0.038
benzyl benzoate	39.01	1765	1759	0.17	ADAMS	0.046	0.002
**Overall**				**92.55**		**91.770**	

***Piper mollicomum* - december/2020**							
Compounds	tR	IRKc	IRKlit	% IN1	IM	Average	SD.
Folha							
α-pinene	6.607	928	932	16.07	ADAMS	11.307	0.021
β-pinene	7.901	941	974	9.60	ADAMS	6.493	0.011
1,8-cineole	9.755	1037	1026	25.80	ADAMS	8.363	0.009
γ-terpinene	10.569	1045	1054	0.43	ADAMS	4.038	0.003
linalool	12.165	1087	1088	4.16	ADAMS	11.287	0.043
ocimene	13.249	1119	1128	0.27	ADAMS	0.421	0.004
α-terpineol	15.04	1150	1162	3.41	ADAMS	3.012	0.002
terpinen-4-ol	15.479	1189	1174	0.58	ADAMS	0.664	0.006
undecanone	20.163	1307	1293	0.18	ADAMS	0.714	0.004
undecanol	20.607	1309	1301	0.24	ADAMS	0.907	0.006
δ-elemene	22.005	1346	1335	1.30	ADAMS	2.179	0.001
α-cubebene	23.468	1347	1348	0.27	ADAMS	0.248	0.001
α-copaene	23.731	1365	1374	0.23	ADAMS	0.428	0.002

β-elemene	24.375	1376	1389	7.66	ADAMS	8.626	0.019
germacrene D	28.073	1495	1480	4.08	ADAMS	1.411	0.004
β-selinene	28.368	1498	1452	2.83	ADAMS	2.342	0.009
E,E-α-farnesene	28.958	1524	1505	1.08	ADAMS	0.977	0.006
δ-cadinene	29.558	1529	1522	1.28	ADAMS	1.944	0.001
Elemol	30.697	1541	1548	0.39	ADAMS	0.326	0.001
E-nerolidol	31.242	1546	1561	3.25	ADAMS	1.760	0.004
caryophyllene oxide	32.017	1594	1582	0.62	ADAMS	0.114	0.023
gleenol	32.183	1595	1586	0.20	ADAMS	0.036	0.001
elemenone	32.507	1596	1589	0.23	ADAMS	0.670	0.006
pogostol	32.889	1645	1651	0.32	ADAMS	0.007	0.000
zerumbone	33.075	1728	1732	0.51	ADAMS	0.054	0.005
caryophyllene acetate	36.658	1804	1417	0.82	ADAMS	0.094	0.002
benzyl benzoate	38.734	1765	1759	0.39	ADAMS	0.106	0.015
**Overall**				**86.20**		**68.527**	
Inflorescence stage 1							
α-pinene	8.834	937	932	1.19	ADAMS	8.582	0.300
sabinene	9.271	954	969	0.37	ADAMS	0.437	0.007
β-pinene	9.499	941	974	3.53	ADAMS	6.388	0.130
myrcene	9.568	943	988	0.97	ADAMS	1.153	0.131
α-phellandrene	9.918	979	1002	0.17	ADAMS	0.740	0.051
α-terpinene	10.158	1000	1014	1.29	ADAMS	0.474	0.082
1,8-cineole	10.838	996	1026	12.47	ADAMS	32.619	0.236
linalool	12.667	1087	1095	4.00	ADAMS	6.112	0.173
ocimene	13.404	1119	1128	0.42	ADAMS	0.308	0.012
δ-terpineol	15.257	1157	1162	1.90	ADAMS	1.753	0.042
terpinen-4-ol	15.45	1189	1174	1.13	ADAMS	0.502	0.012
α-terpineol	15.964	1150	1162	9.44	ADAMS	3.956	0.097
α-copaene	23.747	1375	1374	0.18	ADAMS	0.072	0.004
β-elemene	24.335	1376	1389	0.50	ADAMS	1.200	0.035
E-caryophyllene	25.795	1415	1417	4.24	ADAMS	1.441	0.013

E,E-α-farnesene	26.894	1524	1505	1.36	ADAMS	0.173	0.002
β-gurjunene	27.821	1525	1431	0.34	ADAMS	0.187	0.005
γ-curcumene	27.897	1528	1515	0.20	ADAMS	0.215	0.018
germacrene D	28.06	1529	1480	0.35	ADAMS	0.112	0.004
β-selinene	28.395	1531	1452	0.46	ADAMS	0.243	0.011
α-cubebene	28.511	1535	1348	0.21	ADAMS	0.140	0.001
α-selinene	28.691	1537	1498	0.52	ADAMS	0.083	0.003
α-muurolene	28.776	1538	1500	0.17	ADAMS	0.209	0.002
β-bisabolene	29.132	1540	1505	0.17	ADAMS	0.138	0.005
α-cedrene	29.197	1541	1410	0.25	ADAMS	0.260	0.002
α-amorphene	29.346	1543	1483	0.15	ADAMS	0.033	0.002
δ-cadinene	29.582	1544	1522	0.91	ADAMS	0.099	0.001
isoledene	29.725	1545	1374	0.17	ADAMS	0.263	0.002
E-nerolidol	31.358	1566	1561	3.41	ADAMS	0.768	0.013
cis-cadina-1,4-diene	33.785	1780	1495	0.26	ADAMS	0.123	0.004
eupatoriochromene	36.771	1798	1761	28.06	ADAMS	15.264	0.318
caryophyllene acetate	37.098	1804	1417	1.78	ADAMS	0.427	0.008
benzyl benzoate	38.831	1808	1759	0.19	ADAMS	1.997	0.249
**Overall**				**80.76**		**86.468**	
Inflorescence stage 2							
α-pinene	8.742	937	932	0.97	ADAMS	7.037	0.976
sabinene	9.199	954	969	0.47	ADAMS	0.386	0.117
β-pinene	9.45	942	932	3.22	ADAMS	6.177	0.908
myrcene	9.52	943	988	0.73	ADAMS	1.653	0.654
α-phellandrene	9.864	979	1002	0.16	ADAMS	0.253	0.033
α-terpinene	10.108	1000	1014	0.41	ADAMS	2.395	1.675
1,8-cineole	10.785	1035	1026	10.52	ADAMS	33.015	0.481
γ-terpinene	11.361	1045	1054	0.59	ADAMS	3.572	0.587
tolualdehyde	11.669	1054	1062	0.65	ADAMS	1.034	0.375
ocimene	13.511	1120	1128	0.55	ADAMS	1.706	0.938
α-terpineol	15.353	1177	1186	9.60	ADAMS	4.842	0.740

terpinen-4-ol	15.762	1189	1174	0.91	ADAMS	0.024	0.001
linalool propanoate	22.078	1322	1334	1.22	ADAMS	0.127	0.017
α-copaene	23.784	1365	1374	0.51	ADAMS	0.106	0.026
β-elemene	24.52	1376	1389	3.46	ADAMS	0.351	0.022
E-caryophyllene	24.909	1415	1417	6.30	ADAMS	2.492	0.232
germacrene D	26.173	1495	1480	7.43	ADAMS	1.420	0.344
α-amorphene	26.993	1497	1483	1.53	ADAMS	0.798	0.117
α-humulene	27.287	1498	1452	2.76	ADAMS	0.852	0.476
δ-selinene	27.807	1500	1492	0.39	ADAMS	0.318	0.007
γ-curcumene	28.028	1528	1515	0.69	ADAMS	0.468	0.025
β-selinene	28.501	1529	1452	0.77	ADAMS	0.746	0.033
tridecanone	28.643	1531	1495	0.29	ADAMS	0.428	0.001
α-selinene	28.797	1500	1498	1.10	ADAMS	0.327	0.027
α-muurolene	28.886	1538	1500	0.71	ADAMS	0.491	0.008
E,E-α-farnesene	29.138	1524	1505	1.68	ADAMS	0.151	0.018
δ-cadinene	29.436	1529	1522	2.06	ADAMS	0.828	0.007
E-iso-γ-bisabolene	30.403	1535	1528	0.15	ADAMS	0.113	0.002
Elemol	30.748	1541	1548	0.40	ADAMS	0.125	0.001
E-nerolidol	31.586	1546	1561	5.56	ADAMS	1.461	0.121
caryophyllene oxide	32.141	1594	1582	0.61	ADAMS	0.119	0.018
α-copaene	34.567	1640	1374	0.77	ADAMS	0.009	0.001
α-humulene	34.852	1643	1452	0.89	ADAMS	0.130	0.003
Viridiflorol	35.042	1651	1592	2.15	ADAMS	0.229	0.008
eupatoriochromene	36.126	1653	1761	14.16	ADAMS	6.547	0.497
caryophyllene acetate	37.007	1661	1417	1.38	ADAMS	0.258	0.040
benzyl benzoate	38.878	1765	1759	0.58	ADAMS	0.085	0.015
**Overall**				**86.33**		**81.073**	
Inflorescence stage 3							
α-pinene	8.865	936	932	1.05	ADAMS	3.503	0.089
β-pinene	9.488	953	974	2.96	ADAMS	8.131	0.012
myrcene	9.553	966	988	0.96	ADAMS	0.919	0.062

α-phellandrene	9.927	988	1002	0.13	ADAMS	0.121	0.013
α-terpinene	10.169	996	1014	0.26	ADAMS	0.127	0.007
1,8-cineole	10.684	1025	1026	6.83	ADAMS	22.927	0.931
γ-terpinene	11.281	1051	1054	2.08	ADAMS	5.550	0.478
tolualdehyde	11.601	1056	1062	0.75	ADAMS	0.554	0.055
terpinolene	12.066	1080	1086	0.42	ADAMS	0.061	0.030
linalool	12.587	1083	1008	3.37	ADAMS	8.461	0.192
ocimene	13.367	1127	1128	1.03	ADAMS	4.352	0.056
α-terpineol	15.827	1163	1162	1.86	ADAMS	1.656	0.034
E,E-α-farnesene	29.021	1521	1505	1.60	ADAMS	0.113	0.016
E-iso-γ-bisabolene	29.138	1533	1528	0.67	ADAMS	0.281	0.019
α-cedrene	29.202	1541	1410	0.31	ADAMS	0.196	0.108
α-amorphene	29.357	1545	1483	0.21	ADAMS	0.261	0.048
δ-cadinene	29.597	1546	1522	1.38	ADAMS	3.310	0.320
E-nerolidol	31.506	1556	1561	7.39	ADAMS	2.659	0.759
caryophyllene oxide	32.08	1595	1496	0.40	ADAMS	0.444	0.015
γ-gurjunene	32.292	1598	1431	0.57	ADAMS	0.225	0.047
ledol	32.963	1603	1602	0.77	ADAMS	0.516	0.001
δ-selinene	33.418	1609	1492	0.64	ADAMS	0.080	0.010
cis-cadina-1,4-diene	33.813	1610	1495	0.42	ADAMS	0.080	0.005
2E,4E-dodecadienal	33.955	1628	1518	0.50	ADAMS	0.216	0.112
α-ylangene	34.023	1630	1373	0.20	ADAMS	0.180	0.013
α-humulene	34.79	1643	1452	2.01	ADAMS	2.496	0.478
Viridiflorol	34.953	1656	1592	1.58	ADAMS	0.163	0.007
eupatoriochromene	36.104	1657	1761	23.60	ADAMS	15.652	0.567
caryophyllene acetate	36.687	1662	1417	0.46	ADAMS	0.375	0.137
benzyl benzoate	38.822	1763	1759	0.32	ADAMS	0.207	0.203
**Overall**				**64.73**		**83.816**	
Inflorescence stage 4							
α-pinene	6.635	939	932	14.79	ADAMS	14.703	0.147
β-pinene	7.921	943	974	9.94	ADAMS	3.190	0.051

1,8-cineole	9.868	1036	1026	34.60	ADAMS	23.076	0.008
γ-terpinene	10.599	1051	1054	0.82	ADAMS	0.034	0.002
sabinene	11.044	1060	969	0.16	ADAMS	0.217	0.001
terpinolene	11.632	1069	1086	0.45	ADAMS	0.263	0.012
linalool	12.187	1075	1098	4.16	ADAMS	4.455	0.007
ocimene	13.26	1121	1128	1.02	ADAMS	0.769	0.002
α-terpineol	15.063	1134	1162	7.44	ADAMS	5.026	0.005
α-selinene	28.644	1505	1498	0.17	ADAMS	0.271	0.015
E,E-α-farnesene	28.939	1524	1505	0.46	ADAMS	0.539	0.004
E-nerolidol	31.166	1546	1561	0.97	ADAMS	0.133	0.008
α-humulene	34.708	1643	1452	0.28	ADAMS	0.247	0.008
Viridiflorol	34.882	1656	1592	0.20	ADAMS	1.230	0.251
eupatoriochromene	35.681	1801	1761	15.95	ADAMS	32.923	0.037
caryophyllene acetate	36.769	1817	1701	1.02	ADAMS	1.131	0.002
**Overall**				**92.43**		**88.208**	
Inflorescence stage 5							
α-pinene	8.857	945	932	1.85	ADAMS	6.952	0.816
sabinene	9.281	957	969	0.33	ADAMS	0.253	0.022
β-pinene	9.459	946	974	3.01	ADAMS	5.515	0.111
myrcene	9.529	947	988	1.05	ADAMS	2.244	0.138
α-phellandrene	9.929	979	1002	0.14	ADAMS	0.652	0.071
α-terpinene	10.174	983	1014	0.37	ADAMS	0.691	0.008
1,8-cineole	10.82	1019	1026	11.29	ADAMS	26.282	0.185
E-β-ocimene	10.986	1039	1044	1.45	ADAMS	0.981	0.020
γ-terpinene	11.321	1055	1054	0.47	ADAMS	0.462	0.018
terpinolene	12.137	1066	1086	0.46	ADAMS	0.142	0.006
linalool	12.934	1096	1098	10.99	ADAMS	10.466	0.328
ocimene	13.549	1123	1128	0.93	ADAMS	0.278	0.005
trans-β-terpineol	15.046	1147	1159	0.59	ADAMS	0.186	0.003
α-terpineol	16.042	1157	1162	4.25	ADAMS	2.116	0.053
β-elemene	24.325	1381	1389	0.39	ADAMS	0.148	0.004

E-caryophyllene	24.789	1415	1417	5.24	ADAMS	2.445	0.057
germacrene D	25.975	1477	1480	0.65	ADAMS	1.185	0.028
α-humulene	26.208	1453	1452	6.64	ADAMS	1.780	0.033
α-amorphene	27.807	1462	1483	0.21	ADAMS	0.173	0.008
γ-curcumene	27.883	1519	1515	0.21	ADAMS	0.257	0.020
β-selinene	28.375	1557	1498	0.36	ADAMS	0.345	0.007
α-selinene	28.669	1517	1522	0.78	ADAMS	0.040	0.002
E,E-α-farnesene	28.954	1507	1505	0.39	ADAMS	0.061	0.001
E-iso-γ-bisabolene	29.099	1535	1528	0.19	ADAMS	0.096	0.003
δ-cadinene	29.552	1520	1522	0.32	ADAMS	0.448	0.042
E-nerolidol	30.3	1554	1561	6.52	ADAMS	2.489	0.010
caryophyllene oxide	30.699	1585	1582	0.65	ADAMS	0.042	0.001
γ-gurjunene	32.925	1590	1409	0.27	ADAMS	0.064	0.003
valencene	33.516	1593	1496	0.11	ADAMS	0.367	0.037
cis-cadina-1,4-diene	33.774	1597	1495	0.57	ADAMS	0.412	0.020
eupatoriochromene	35.91	1697	1761	21.07	ADAMS	9.651	0.203
caryophyllene acetate	36.861	1700	1701	1.82	ADAMS	0.581	0.007
benzyl benzoate	38.766	1763	1759	0.29	ADAMS	1.028	0.102
**Overall**				**83.57**		**78.832**	

***Piper mollicomum* - january/2021**							
Compounds	tR	IRKc	IRKlit	% IN1	IM	Average	SD.
Folha							
α-pinene	6.539	928	932	11.94	ADAMS	3.799	0.002
β-pinene	7.913	941	974	11.08	ADAMS	1.940	0.002
1,8-cineole	9.811	1037	1026	28.19	ADAMS	11.244	0.010
γ-terpinene	10.587	1045	1054	0.60	ADAMS	0.036	0.001
linalool oxide <cis->	11.042	1053	1067	0.23	ADAMS	0.236	0.045
terpinolene	11.625	1071	1086	0.32	ADAMS	0.484	0.012
linalool	12.127	1087	1088	1.34	ADAMS	2.158	0.007

ocimene	13.255	1119	1128	0.30	ADAMS	0.118	0.000
α-terpineol	15.045	1150	1162	4.76	ADAMS	9.013	0.011
terpinen-4-ol	15.487	1189	1174	0.81	ADAMS	7.328	0.008
α-copaene	23.733	1365	1374	0.20	ADAMS	0.586	0.005
β-elemene	24.382	1376	1389	4.23	ADAMS	11.551	0.024
9-epi-E-caryophyllene	25.586	1415	1464	4.51	ADAMS	3.538	0.001
germacrene D	25.998	1495	1480	4.40	ADAMS	1.463	0.002
α-humulene	27.054	1498	1452	2.13	ADAMS	1.283	0.008
α-amorphene	27.826	1523	1483	1.48	ADAMS	0.884	0.002
Cubebene <β->	28.076	1456	1387	2.95	ADAMS	0.437	0.060
β-selinene	28.368	1531	1452	0.42	ADAMS	0.850	0.031
tridecanone	28.557	1526	1495	0.20	ADAMS	0.538	0.051
germacrene B	28.665	1516	1559	1.34	ADAMS	6.082	0.013
E,E-α-farnesene	28.946	1524	1505	0.57	ADAMS	1.345	0.003
δ-cadinene	29.328	1529	1522	0.98	ADAMS	0.808	0.001
Elemol	30.697	1541	1548	0.46	ADAMS	1.880	0.001
E-nerolidol	31.208	1546	1561	2.11	ADAMS	2.448	0.007
spathulenol	31.824	1554	1577	0.25	ADAMS	1.113	0.006
caryophyllene oxide	32.016	1594	1582	0.72	ADAMS	1.889	0.059
γ-gurjunene	32.174	1600	1475	0.21	ADAMS	0.220	0.057
ledol	32.885	1603	1602	0.29	ADAMS	0.073	0.000
zerumbone	33.074	1728	1732	0.61	ADAMS	0.739	0.024
Santalol acetate <(Z)-α->	33.304	1765	1777	0.63	ADAMS	0.034	0.007
α-humulene	34.73	1794	1452	0.64	ADAMS	0.034	0.004
α-muurolol	34.805	1800	1640	0.75	ADAMS	0.077	0.002
Viridiflorol	34.899	1651	1592	0.54	ADAMS	0.017	0.003
eupatoriochromene	35.344	1798	1761	0.59	ADAMS	0.027	0.002
caryophyllene acetate	36.662	1804	1417	0.98	ADAMS	0.146	0.005
benzyl benzoate	38.733	1765	1759	0.39	ADAMS	0.142	0.016
**Overall**				**92.15**		**74.564**	
Inflorescence stage 1							

α-pinene	8.922	937	932	3.12	ADAMS	12.957	0.017
β-pinene	9.501	941	974	5.09	ADAMS	15.845	0.081
myrcene	9.561	943	988	1.72	ADAMS	3.274	0.117
α-phellandrene	9.959	979	1002	0.13	ADAMS	0.129	0.007
α-terpinene	10.201	1000	1014	0.55	ADAMS	1.373	0.005
1,8-cineole	10.809	996	1026	19.38	ADAMS	13.510	0.294
γ-terpinene	11.292	1045	1054	0.86	ADAMS	11.002	0.012
terpinolene	12.078	1071	1086	0.56	ADAMS	10.541	0.143
linalool	12.507	1087	1095	2.89	ADAMS	2.432	0.059
trans-sabinene hydrate	12.608	1089	1098	0.21	ADAMS	0.363	0.049
ocimene	13.391	1119	1128	0.29	ADAMS	3.181	0.189
δ-terpineol	15.174	1157	1162	1.38	ADAMS	0.166	0.027
terpinen-4-ol	15.599	1189	1174	0.81	ADAMS	0.058	0.031
α-terpineol	15.847	1150	1162	9.35	ADAMS	1.212	0.237
β-elemene	24.292	1376	1389	0.16	ADAMS	0.061	0.002
E-caryophyllene	25.554	1415	1417	1.44	ADAMS	0.191	0.021
α-humulene	26.841	1498	1452	1.11	ADAMS	0.161	0.006
E-nerolidol	31.154	1566	1561	1.24	ADAMS	0.136	0.002
eupatoriochromene	35.646	1798	1761	19.81	ADAMS	2.979	0.194
**Overall**				**70.10**		**79.570**	
Inflorescence stage 2							
α-pinene	8.914	937	932	1.14	ADAMS	13.587	0.003
β-pinene	9.48	942	932	3.95	ADAMS	18.189	0.050
myrcene	9.542	943	988	1.24	ADAMS	2.661	0.040
α-terpinene	10.198	1000	1014	0.33	ADAMS	0.094	0.004
1,8-cineole	10.779	1035	1026	21.04	ADAMS	16.503	0.123
linalool	12.585	1083	1008	7.82	ADAMS	10.342	0.026
ocimene	13.389	1120	1128	0.23	ADAMS	0.076	0.001
δ-terpineol	15.181	1137	1162	1.94	ADAMS	1.921	0.069
α-terpineol	16.301	1177	1186	12.54	ADAMS	2.243	0.252
E-nerolidol	31.174	1546	1561	1.99	ADAMS	0.124	0.001

E,E-α-farnesene	33.855	1521	1505	0.19	ADAMS	0.050	0.001
α-humulene	34.687	1643	1452	0.50	ADAMS	0.033	0.009
eupatoriochromene	35.411	1653	1761	4.19	ADAMS	0.127	0.003
caryophyllene acetate	36.642	1661	1417	0.91	ADAMS	0.043	0.001
benzyl benzoate	38.702	1765	1759	0.15	ADAMS	0.382	0.027
**Overall**				**58.16**		**66.374**	
Inflorescence stage 3							
α-pinene	8.846	936	932	2.45	ADAMS	2.252	0.268
sabinene	9.289	950	969	0.60	ADAMS	1.072	0.020
β-pinene	9.561	953	974	5.76	ADAMS	2.421	0.474
myrcene	9.636	966	988	1.56	ADAMS	1.548	0.181
α-phellandrene	9.948	988	1002	0.11	ADAMS	1.414	0.390
α-terpinene	10.183	996	1014	0.59	ADAMS	1.539	0.383
1,8-cineole	10.986	1025	1026	23.98	ADAMS	33.752	0.268
linalool	12.908	1083	1008	5.10	ADAMS	21.600	1.435
trans-sabinene hydrate	12.964	1089	1098	0.16	ADAMS	0.713	0.013
ocimene	13.265	1127	1128	1.04	ADAMS	0.105	0.004
δ-terpineol	15.329	1137	1162	2.06	ADAMS	1.530	0.125
bornyl acetate	15.429	1286	1284	0.82	ADAMS	0.015	0.001
α-terpineol	15.895	1163	1162	9.82	ADAMS	3.316	0.206
β-elemene	24.349	1381	1389	0.32	ADAMS	0.079	0.009
E-caryophyllene	24.997	1419	1417	3.08	ADAMS	0.126	0.009
germacrene D	26	1496	1480	0.43	ADAMS	0.128	0.016
α-humulene	27.199	1498	1452	2.07	ADAMS	1.141	0.091
γ-curcumene	27.91	1491	1515	0.17	ADAMS	0.127	0.016
β-selinene	28.397	1500	1489	0.20	ADAMS	0.143	0.055
α-selinene	28.69	1502	1498	0.24	ADAMS	0.058	0.002
E,E-α-farnesene	29.345	1521	1505	0.29	ADAMS	0.060	0.002
δ-cadinene	29.564	1546	1522	0.17	ADAMS	0.128	0.004
E-nerolidol	31.314	1556	1561	1.52	ADAMS	0.034	0.007
caryophyllene oxide	32.048	1595	1496	0.26	ADAMS	0.012	0.000

zerumbone	33.105	1728	1732	0.30	ADAMS	0.035	0.004
γ-gurjunene	33.384	1598	1431	0.69	ADAMS	0.015	0.002
eupatoriochromene	36.104	1657	1761	20.58	ADAMS	4.574	0.288
α-humulene	34.779	1643	1452	1.88	ADAMS	0.243	0.015
caryophyllene acetate	37.018	1662	1417	1.41	ADAMS	0.496	0.053
**Overall**				**87.66**		**78.675**	
Inflorescence stage 4							
β-pinene	7.877	943	974	3.73	ADAMS	2.492	0.464
1,8-cineole	9.779	1036	1026	18.11	ADAMS	49.322	1.380
ocimene	10.097	1121	1128	2.19	ADAMS	1.366	0.389
γ-terpinene	10.554	1051	1054	0.22	ADAMS	0.029	0.005
terpinolene	11.597	1080	1086	0.21	ADAMS	2.718	0.866
linalool	12.363	1083	1008	9.21	ADAMS	7.541	0.720
ocimene	13.252	1127	1128	0.61	ADAMS	0.352	0.033
bornyl acetate	15.474	1286	1284	0.51	ADAMS	0.431	0.049
α-terpineol	16.237	1134	1162	4.71	ADAMS	3.538	0.542
E-caryophyllene	25.566	1415	1417	1.24	ADAMS	0.813	0.077
α-humulene	27.027	1487	1452	1.14	ADAMS	0.331	0.033
β-selinene	28.342	1500	1489	0.40	ADAMS	0.143	0.034
α-selinene	28.633	1505	1498	0.17	ADAMS	0.143	0.031
E,E-α-farnesene	28.919	1524	1505	0.16	ADAMS	0.265	0.055
δ-cadinene	29.517	1546	1522	0.15	ADAMS	0.262	0.047
E-nerolidol	31.273	1546	1561	4.23	ADAMS	1.703	0.174
caryophyllene oxide	32.001	1595	1496	0.16	ADAMS	0.016	0.002
δ-cadinene	34.255	1546	1522	0.13	ADAMS	0.048	0.006
α-copaene	34.432	1798	1374	0.14	ADAMS	0.016	0.004
α-muurolol	34.763	1800	1640	0.16	ADAMS	0.062	0.011
Viridiflorol	34.865	1656	1592	0.32	ADAMS	0.154	0.021
eupatoriochromene	35.518	1801	1761	7.78	ADAMS	3.710	0.367
caryophyllene acetate	36.646	1817	1701	0.48	ADAMS	0.202	0.058
**Overall**				**56.16**		**75.656**	

Inflorescence stage 5							
α-pinene	8.758	935	932	1.66	ADAMS	4.705	2.173
β-pinene	9.518	949	974	4.97	ADAMS	6.011	1.368
α-terpinene	10.142	998	1014	0.16	ADAMS	0.217	0.017
1,8-cineole	10.961	1022	1026	17.37	ADAMS	47.722	3.531
E-β-ocimene	11.571	1040	1044	0.97	ADAMS	0.887	0.046
γ-terpinene	11.745	1057	1054	0.52	ADAMS	0.026	0.005
terpinolene	12.359	1071	1086	0.37	ADAMS	0.109	0.140
linalool	13.403	1091	1098	13.03	ADAMS	6.776	1.667
ocimene	13.977	1129	1128	1.25	ADAMS	0.196	0.015
camphor	14.61	1149	1141	0.15	NIST	0.157	0.077
trans-β-terpineol	15.392	1155	1159	1.00	ADAMS	0.385	0.047
α-terpineol	17.002	1168	1162	8.18	ADAMS	4.168	0.603
E-caryophyllene	25.134	1415	1417	1.41	ADAMS	0.704	0.056
α-humulene	25.79	1441	1452	5.29	ADAMS	1.190	0.122
E-nerolidol	30.618	1561	1561	5.98	ADAMS	0.280	0.061
α-selinene	33.567	1601	1498	0.45	ADAMS	0.201	0.056
cis-cadina-1,4-diene	33.946	1653	1495	0.32	ADAMS	0.788	0.030
eupatoriochromene	36.109	1757	1761	11.38	ADAMS	2.369	0.486
caryophyllene acetate	37.122	1761	1701	2.50	ADAMS	0.243	0.065
benzyl benzoate	38.88	1766	1759	0.37		0.159	0.063
**Overall**				**76.96**		**77.291**	

tR = Calculated retention index (column HP-5MS); IRlit = Literature retention index (Adams. 2009); SD - standard deviation; IM - identification methodology ;All substances were identified by EM and DIC according to the experimental.

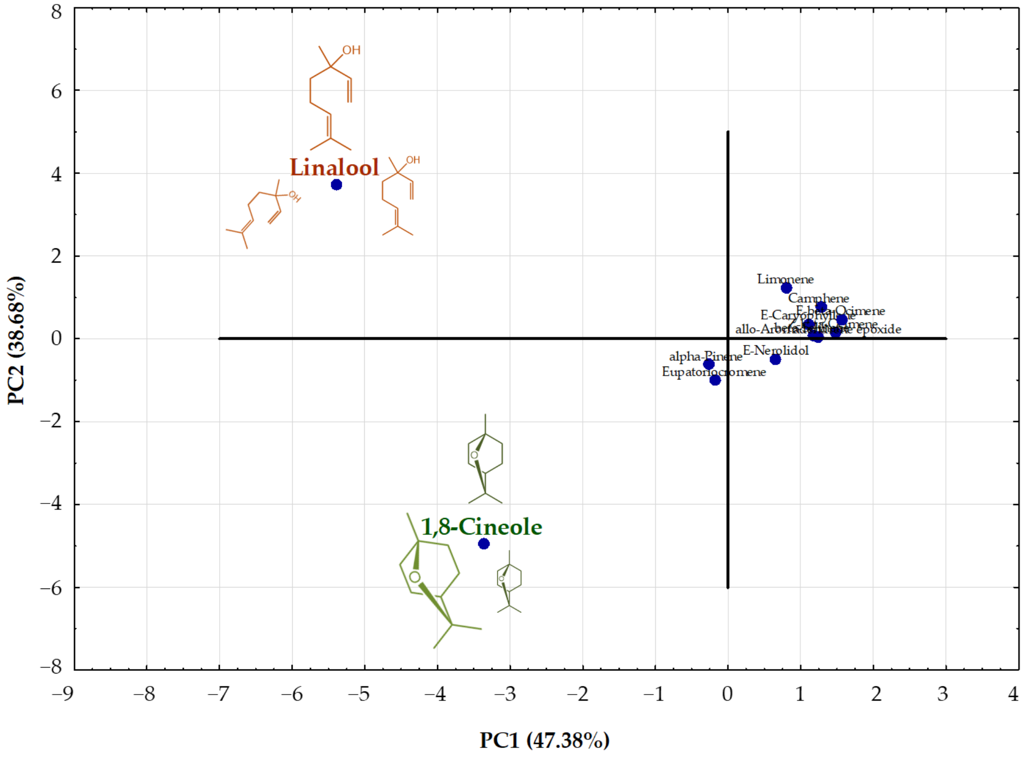

Figure S1. Ordering diagram produced by the principal component analysis (PCA) demonstrating the projections of the constituents present in the EOs from leaves and inflorescences of *Piper mollicomum* Kunth in the months under study (September 2020 to January 2021).

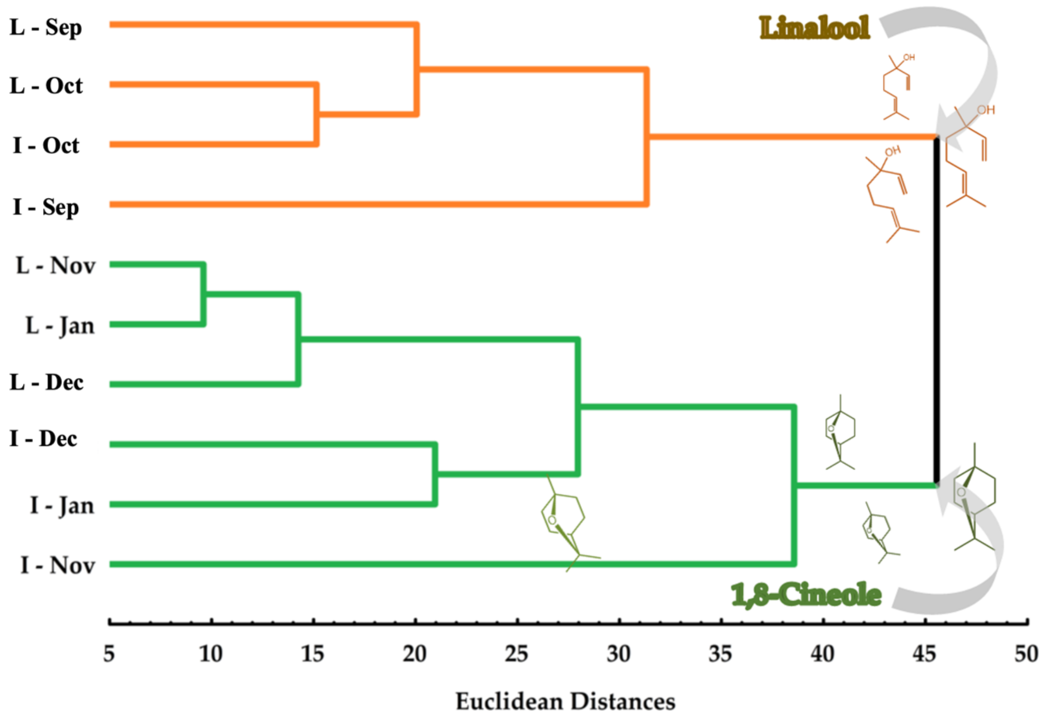

Figure S2. Euclidean Hierarchical Grouping that shows the projections of the constituents present in the EOs from leaves and inflorescences of *Piper mollicomum* Kunth in the months under study (September 2020 to January 2021). Sep – September; Oct – October; Nov – November; Dec – December; Jan – Jan. L – Leaves; I – Inflorescences.

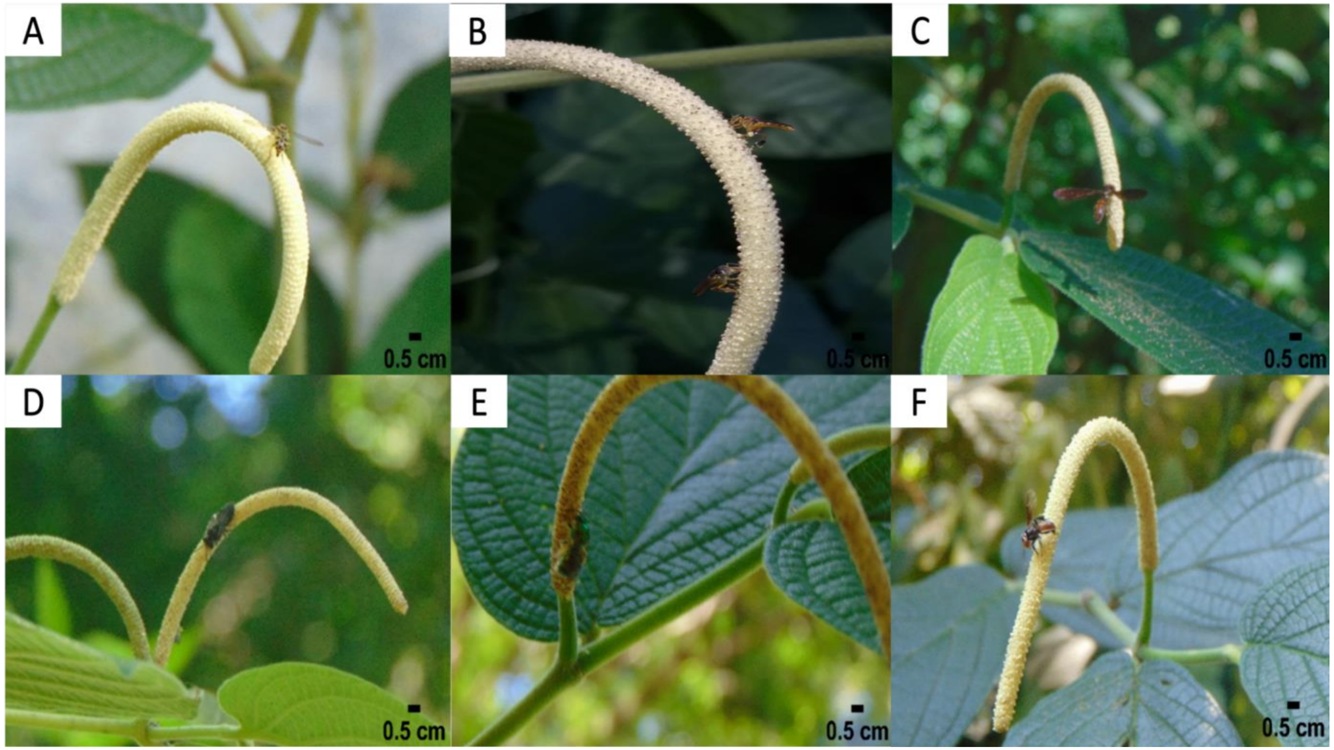

Figure S3. Main visitors of inflorescences of *Piper mollicomum* Kunth from Floresta da Tijuca / RJ. Observations were made weekly from September 2020 to January 2021. A) - Insect 1 (Diptera - *Syrphidae* sp. 1); (B) - Insect 2 (Hymenoptera - *Tetragonisca angustula* Latreille. 1811); (C) - Insect 3 (Diptera - *Syrphidae* sp. 2); (D) - Insect 4 (Hymenoptera - *Halictidae* sp. 1); (E) - Insect 5 (Hymenoptera - *Halictidae* sp. 2); (F) – Insect 6 (Hymenoptera - *Colletidae* sp.).
